# East Texas Medico-Chirurgical Society

**Published:** 1903-10

**Authors:** E. B. Parsons, E. E. Guinn

**Affiliations:** President; Jacksonville, Texas; Secretary; Jacksonville, Texas


### East Texas Medico-Chirurgical Society.

JACKSONVILLE, TEXAS, October 4, 1903

*Texas Medical Journal, Austin, Texas.*

DEAR DOCTOR: Please say in your next issue that the East Texas Medico-Chirurgical Society will meet in Palestine, Texas, on

November 19th and 20th, and you may always expect a full program and splendid entertainment. The Palestine M. D.'s anticipate your wants.

E. B. Parsons, M. D., President.

E. E. Guinn, Secretary.

